# Aphid Colonization Affects Potato Root Exudate Composition and the Hatching of a Soil Borne Pathogen

**DOI:** 10.3389/fpls.2018.01278

**Published:** 2018-09-06

**Authors:** Grace A. Hoysted, Christopher A. Bell, Catherine J. Lilley, Peter E. Urwin

**Affiliations:** Centre for Plant Sciences, University of Leeds, Leeds, United Kingdom

**Keywords:** aboveground–belowground interactions, aphids, fructose, glucose, plant-parasitic nematodes, root exudates

## Abstract

Plants suffer multiple, simultaneous biotic threats from both above and below ground. These pests and/or pathogens are commonly studied on an individual basis and the effects of above-ground pests on below-ground pathogens are poorly defined. Root exudates from potato plants (*Solanum tuberosum* L.) were analyzed to characterize the top-down plant-mediated interactions between a phloem-sucking herbivore (*Myzus persicae*) and a sedentary, endoparasitic nematode (*Globodera pallida*). Increasing inocula of the aphid, *M. persicae*, reduced the root mass of potato plants. Exudates collected from these roots induced significantly lower hatching of second-stage juveniles from *G. pallida* eggs over a 28-day period, than those from uninfested control plants. Inhibition of hatch was significantly positively correlated with size of aphid inoculum. Diminished hatching was partially recovered after treatment with root exudate from uninfested potato plants indicating that the effect on hatching is reversible but cannot be fully recovered. Glucose and fructose content was reduced in root exudates from aphid-infested potato plants compared to controls and these sugars were found to induce hatching of *G. pallida*, but not to the same degree as potato root exudates (PRE). Supplementing aphid-infested PRE with sugars did not recover the hatching potential of the treatment, suggesting that additional compounds play an important role in egg hatch. The first gene upregulated in the closely related potato cyst nematode *Globodera rostochiensis* post-exposure to host root exudate, *Neprilysin-1*, was confirmed to be upregulated in *G. pallida* cysts after exposure to PRE and was also upregulated by the sugar treatments. Significantly reduced upregulation of *Gpa-nep-1* was observed in cysts treated with root exudates from potato plants infested with greater numbers of aphids. Our data suggest that aphid infestation of potato plants affects the composition of root exudates, with consequential effects on the hatching and gene expression of *G. pallida* eggs. This work shows that an above-ground pest can indirectly impact the rhizosphere and reveals secondary effects for control of an economically important below-ground pathogen.

## Introduction

Plants are a primary source of nutrition for a wide range of organisms and are often subject to simultaneous attack from both above and below the ground (Wondafrash et al., 2013; Van Dam et al., 2018). Pest and/or pathogen attack can change the plant’s phenotype, subsequently altering the attraction, behavior, performance, and abundance of other organisms on the same host (Sun et al., 2016). Interactions between spatially separated biota can be mediated systemically, as a result of a tight physiological integration of roots and shoots throughout the plant (Biere and Goverse, 2016).

Plant-feeding aphids and plant-parasitic nematodes (PPN) can be linked through host-mediated interactions (Kaplan et al., 2008; Kutyniok and Müller, 2012; Hoysted et al., 2017). Aphids use their stylet-like mouthparts to feed on photoassimilates found in the host’s sap (Pollard, 1973; Blackman and Eastop, 2000). While feeding, aphids produce a gelling saliva that covers the stylet with a protective sheath and a watery saliva that is secreted into plant cells and the phloem (Miles, 1999; Tjallingii, 2006). Both salivas contain different proteins (Harmel et al., 2008; Hogenhout and Bos, 2011), which can induce or suppress plant defense responses (de Vos et al., 2007; Bos et al., 2010). If aphids are present in high populations, substantial reductions in yield can be observed (Kolbe, 1970) and the transmission of viral diseases by aphids can impose additional stresses (Dixon and Kindlmann, 1998; Foster et al., 2000). Nematodes constitute one of the most abundant phyla of the rhizosphere and many are phytophagous, feeding on the roots of plants (Jones et al., 2013; van Dam and Bouwmeester, 2016; Hewezi and Baum, 2017). Cyst nematodes, such as *Globodera pallida*, are a group of highly evolved sedentary endoparasites that are pathogens of temperate, subtropical, and tropical plant species (Nicol et al., 2011; Cotton et al., 2014). Second-stage juveniles (J2s) hatch in the soil in response to host root exudate, penetrate the root, and migrate intracellularly toward the vascular cylinder where each individual chooses an initial cell from which to form a highly metabolically active feeding site, termed a syncytium, from which the nematode extracts host resources (Lilley et al., 2005; Jones et al., 2013). At maturity the female is fertilized, her body swells, and the cuticle hardens to form a protective cyst that contains hundreds of eggs (Bohlmann, 2015; Moens et al., 2018).

Although aphids and cyst nematodes can share the same host, their attack on the plant is spatially separated: nematodes infect the roots of a suitable host, whereas aphids colonize above-ground biomass (Emden, 1969). The majority of studies on plant-mediated interactions between shoot herbivores and root-parasitic nematodes predominantly focuses on nematode-induced effects on herbivores rather than herbivore-induced effects on nematodes (Van Dam et al., 2005, 2018; Kaplan et al., 2008; Hofmann et al., 2010; Hong et al., 2010; Hol et al., 2013; Wondafrash et al., 2013; Hoysted et al., 2017). Although not as numerous, there have been examples of leaf feeding insects influencing the performance of PPN, however, feeding strategy of the above-ground pest played a role in the outcome of these interactions. Leaf-chewing herbivores (e.g., caterpillars) increased the abundance of PPN; however, sap-feeding insects (e.g., aphids) had a negative impact on the number of PPN present on tobacco (Kaplan et al., 2009). The specialist aphid, *Brevicoryne brassicae* had a negative effect on the abundance of the beet-cyst nematode *Heterodera schachtii* on *Arabidopsis thaliana*, with impaired development of *H. schachtii* possibly attributed to a significant reduction in individual glucosinolates in the roots (Kutyniok and Müller, 2012). Although top-down plant-mediated interactions between aphids and nematodes have been reported, these studies have focused on the indirect effects that above-ground pests can have on nematodes only after the nematode has parasitized its host. To our knowledge, no studies have elucidated the effects of aphids on the composition of plant root exudates and how these exudates may affect PPN.

Plants secrete a large array of compounds into the rhizosphere to facilitate interactions with their biotic environment (van Dam and Bouwmeester, 2016). The presence of certain compounds, termed hatching factors (Devine et al., 1996), in plant root exudates have been reported to stimulate the hatch of cyst nematode eggs from within their protective cysts (Perry, 1997). Hatching factors appear to alter the permeability of the eggshell membrane, causing trehalose to leak from the egg and water to move inward, resulting in rehydration of the J2 and contributing to the eclosion of the nematode (Perry and Beane, 1989). The hatching of some cyst nematodes displays a degree of host specificity, possibly mediated through differences in the structure of certain hatching factors, such as glycinoeclepin A in soybean (*Glycines max*) (Masamune et al., 1982) and solanoeclepin A in tomato and potato (*Solanum lycopersicum* and *S. tuberosum*, respectively) (Schenk et al., 1999). However, hatching of cyst nematodes (*Heterodera* and *Globodera* spp.) is probably much more complex than a simple reliance on a specific compound, as other chemicals such as picloronic acid, sodium thiocyanate, alpha-solanine, and alpha-chaconine (Byrne et al., 2001) can also stimulate hatch. In addition, spontaneous hatch for both *Heterodera* and *Globodera* spp. can occur in the absence of a suitable host crop (Been et al., 1995; Turner and Rowe, 2006). The compounds required for nematode hatch and the mechanisms behind eclosion remain poorly characterized. Additionally, the majority of genes involved in the hatching response has not been uncovered, however, a *G. rostochiensis* neprilysin gene (*Gro-nep-1*) was identified as the first transcript to be upregulated in eggs treated with host root exudate (Duceppe et al., 2017).

The compounds that are exuded by plant roots have been shown to change following attack by above-ground pests and/or pathogens (Rudrappa et al., 2008; Lakshmanan et al., 2012; Neal et al., 2012). Here, we investigated plant-mediated interactions between the generalist aphid *Myzus persicae* and the potato cyst nematode *Globodera pallida* by analyzing root exudates emitted from the potato crop (*Solanum tuberosum* cv. Désirée). Only a few studies have demonstrated the top-down effects of aphids on nematodes (Kutyniok and Müller, 2012, 2013), however, these focused on secondary metabolite changes in the plant caused by the above-ground herbivory. Using a combination of physiological, biochemical, and molecular techniques, we test the hypothesis that systemic changes in root exudates of the potato caused by the presence of *M. persicae* indirectly affect the hatching of *G. pallida* eggs. We describe the composition of sugars contained within these exudates following aphid feeding and investigate the expression response of *Gpa-nep-1*, to study its link to hatching activity.

## Materials and Methods

### Maintenance of Plants, Aphids, and Nematodes

Tuber cuttings of potato (*Solanum tuberosum* L. cv. Désirée) with one chit present were planted in 18 cm pots containing a mix of sand and loam topsoil (50:50). Growth took place in a glasshouse at 20–22°C under 16-h/8-h light/dark cycles for a total period of 3 weeks. Plants were watered every second day. Nymphs of the peach-potato aphid (*Myzus persicae* Sulzer) were obtained from the James Hutton Institute, Invergowrie, Dundee, Scotland. About 10 aphids, which were asexual clones of a wild population originally isolated in Scotland, and subsequently maintained on *S. tuberosum* in containment (Kasprowicz et al., 2008) were transported on leaves of *S. tuberosum* to Leeds in March 2017. Aphid colonies were maintained on potato plants, grown as described above, inside a mesh cage in a containment glasshouse. Cysts of *G. pallida* were extracted from soil of pure stock cultures using the Fenwick’s (1940) method and stored dry at 4°C.

### Preparation of Potato Root Exudates

The 11-day-old potato plants grown from chitted tubers in 50:50 sand/loam mix were infested with either 5, 50, 100, or 200 apterous (wingless) aphids 10 days prior to root harvest. No aphids were released on non-infested control plants. Each set (four plants per set) of aphid-infested plants and non-infested control plants was maintained inside a separate mesh cage to ensure there was no contamination across experiments. Roots of 3-week-old potato plants were excised intact from the bottom of the plant stem and washed to remove excess soil. Excised roots were soaked (80 g per liter tap water) in darkness for 24 h at 4°C. The resulting potato root exudate (PRE) was filter sterilized (0.22 μm) and stored at 4°C. PRE used in the hatching assays was combined from whole root systems obtained from four separate potato plants for each treatment or control.

### Sugar Quantification in Root Exudates

Exudates were prepared from four individual root systems to provide four biological replicates per aphid treatment or control. The concentrations of glucose and fructose in the root exudates were quantified colorimetrically at 340 nm using Glucose (HK) and Fructose assay kits, respectively (Sigma–Aldrich, United States) according to the manufacturer’s instructions provided with the kit. Each of the four biological replicate exudates from the five different treatments was assayed in technical triplicate to provide a mean concentration per replicate that was used for subsequent statistical analysis. Water was a negative control in each assay. Standards provided with the kits were used to construct calibration curves, to convert absorbance readings into μg/ml of glucose and fructose.

### Hatching Assays

For each of the three experiments batches of five cysts (*G. pallida*; 10 replicates per treatment) were placed in wells of 12-well polypropylene plates. One milliliter of PRE from aphid infested plants, control potato plants or sugar solutions was added to each well ensuring the cysts were covered. All three cyst experiments were incubated at 20°C for the duration of the experiment. In the first experiment, PRE from aphid-infested plants was replaced with fresh PRE, and the number of hatched J2s was counted, every 4 days. After 18 days, the same cysts were washed and re-incubated in non-infested control PRE. In a second separate experiment, cysts which had been incubated in aphid-infested PRE were, after 18 days, washed and re-incubated in sugar replacement solutions. Sugar replacement solutions were prepared by adding glucose or fructose to each aphid-infested PRE to bring the concentrations equivalent to those found in non-infested control PRE (16.4 μg/ml glucose and 35.0 μg/ml fructose). Counting of hatched J2s for both the first and second experiment continued until day 28 when emergence of J2s had significantly declined in all treatments. In a third experiment, *G. pallida* cysts were treated with solutions of glucose (16.4 μg/ml), or fructose (35.0 μg/ml) or a combination of the two sugars at those concentrations for 28 days to assess the effect of sugars on *G. pallida* hatching. Cysts incubated in water provided a negative control and PRE was used as a positive control. At the end of each hatching experiment, cysts were opened and the numbers of unhatched J2s were counted, in order to express the data as a percentage of total potential hatch.

### Analysis of *Gpa-nep-*1 Gene Expression

Groups of 10 *G. pallida* cysts (four reps per treatment) were treated with either root exudates from control or aphid-infested plants, sugar solutions, or water for 8 days. Total RNA was prepared using the E.Z.N.A^®^. Plant RNA Kit (Omega Biotek, United States) including a DNase treatment. First-strand cDNA was synthesized from 500 ng RNA using iScript cDNA Synthesis Kit (BioRad, United States) following the manufacturer’s instructions. Quantitative reverse transcriptase (qRT)-PCR was carried out on the resulting cDNA using SsoAdvanced^TM^ Universal SYBR^®^ Green Supermix (BioRad) and a CFX Connect instrument (BioRad, United States). Expression of *G. pallida neprilysin-1* (GPLIN_000276000) was studied and normalized to the housekeeping gene *Elongation Factor 1*-α (Nicot et al., 2005). Primers Gpnep1F (5′-TCACGGCATCAGACAACATT-3′), Gpnep1R (5′-CCGTGTCACTTAGCCGATTT-3′), GpEF1aF (5′-AATGACCCGGCAAAGGAGA-3′), and GPEF1aR (5′-GTAGCCGGCTGAGATCTGTC-3′) were used for analysis of *G. pallida neprilysin-1* and *Elongation Factor 1*-α, respectively. Control reactions contained water instead of template. Each primer pair had an amplification efficiency of 97–101% and *r*^2^ correlation coefficients for standard curves ranged between 0.94 and 0.99. Primer pair efficiencies were calculated using the BioRad CFX Manager 3.1 software. Gene expression analysis was performed on four biological replicates for all treatments and each reaction was carried out in triplicate. *C*_T_ values were determined using the BioRad CFX Manager 3.1 software. Relative expression between treatments was determined using the 2^-ΔΔ*C*_T_^ method as described in Livak and Schmittgen (2001).

### Data Analysis

One-way ANOVA and Student-Newman-Keuls (SNK) *post hoc* tests were used to determine the significance of differences in potato root weight, final percentage hatch, sugar content of root exudates and gene expression data. All data were checked for normality using the Shapiro–Wilks test prior to statistical analysis. Pearson’s correlation was used to measure the strength and direction of the relationship between inhibition of nematode hatch and size of aphid inoculum. SPSS v24 (IBM Corporation Armonk, New York, NY, United States) was used for all statistical analysis.

## Results

### Increased Inoculum of *Myzus persicae* Reduces Below-Ground Tissue in Potato Plants

**FIGURE 1:**
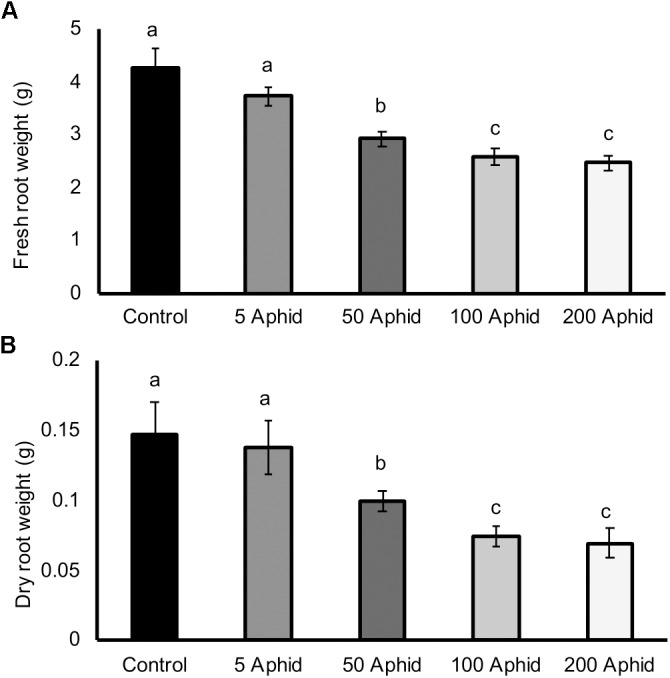
Effect of *Myzus persicae* inoculum on the fresh **(A)** and dry **(B)** weight of potato roots (*Solanum tuberosum* cv. Désirée) 10 dpi. Values are means ± SEM from at least four replicates with different letters indicating significant differences between treatments (*P* < 0.01).

There was a significant reduction in both the fresh and dry root weights of potato plants that had been infested with at least 50 *Myzus persicae* individuals for 10 days compared to the roots of non-infested potato plants (**Figures 1A,B**; *P* ≤ 0.05). Increasing inocula of aphids resulted in greater reductions in both fresh and dry weights of roots (**Figures 1A,B**; *P <* 0.05), with a significant dose-dependent correlation (Pearson’s coefficient of *r* = -0.727, *P* < 0.01).

### Root Exudate From Aphid-Infested Potato Plants Induces Diminished Hatching of *Globodera pallida*

**FIGURE 2:**
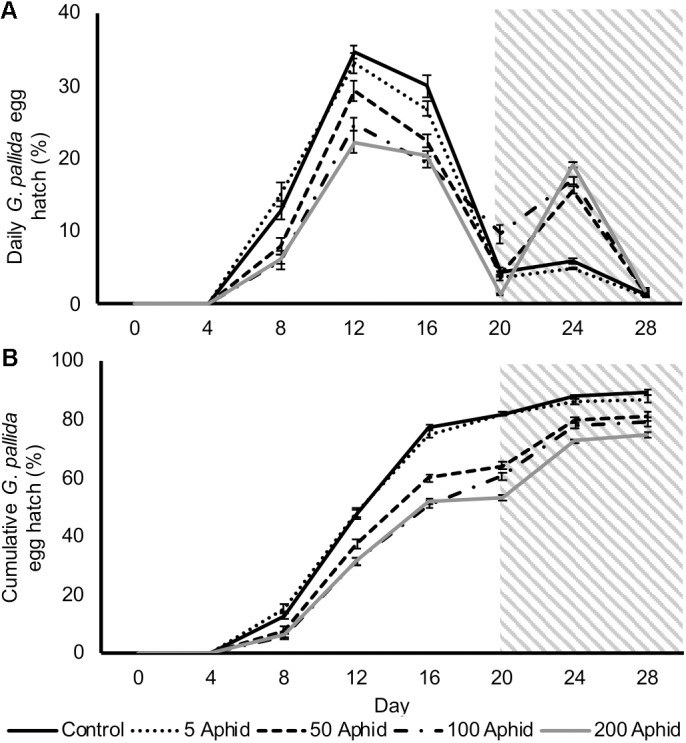
Daily **(A)** and cumulative **(B)** *Globodera pallida* percentage egg hatch from cysts treated with root exudate from non-infested control and *Myzus persicae* infested potato plants (days 0–20). Initial inoculums of 5, 50, 100, and 200 aphids were applied to the leaves of potato plants for 10 days before collection of exudate. All cysts were treated with root exudate from non-infested potato plants (control) at day 20–28 (indicated by gray box). Values are means ± SEM from 10 replicates with five cysts per replicate.

In this study, we investigated the possible indirect effect that aphids may have on cyst nematodes via root exudate. Hatching of *G. pallida* was significantly reduced when cysts were incubated in PRE from potato plants infested with > 5 *M. persicae* compared to exudates from non-infested control plants (**Figures 2A,B**; *P* < 0.05). There was a significant positive correlation between the aphid inoculum level and the reduction of *G. pallida* hatching over 28 days (**Figures 2A,B**; Pearson’s correlation *r* = -0.792, *P* < 0.01). Diminished hatching was partially recovered on day 20 after treatment with root exudate from uninfected potato plants, resulting in a second peak of hatching (**Figure 2A**). This indicates that the effect on hatching is reversible, however, hatching was not fully recovered to PRE control treatment levels (**Figure 2B**).

### Increasing Inoculum of *M. persicae* Results in a Decreased Glucose and Fructose Content in Potato Root Exudates

**FIGURE 3:**
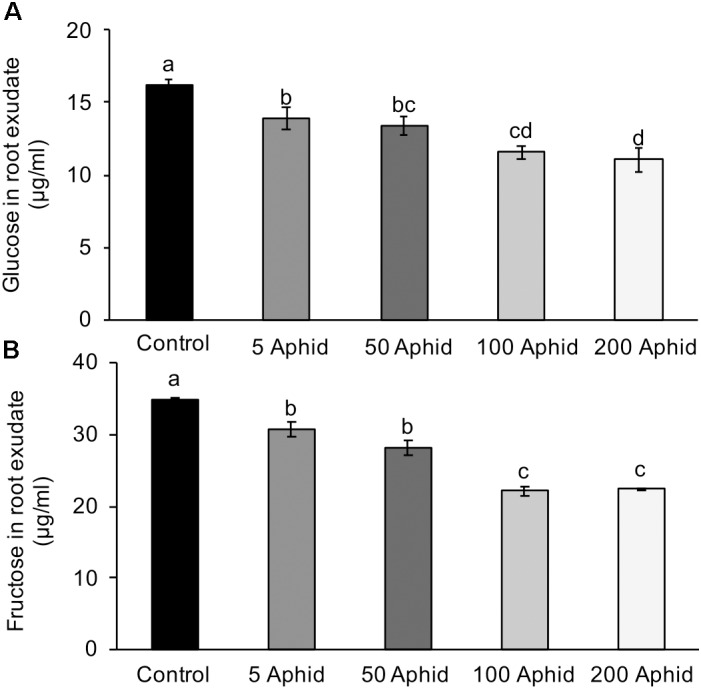
Glucose **(A)** and fructose **(B)** content in root exudates from control and *Myzus persicae* infested potato plants. Values are means ± SEM at least four replicates with different letters denoting significance (*P* < 0.05 one-way ANOVA and SNK).

Sugars are present in the honeydew of *M. persicae* implicating aphids in the translocation of sugars around the host plant (Hussain et al., 1974), therefore, we analyzed the amounts of glucose and fructose present in the control and treatment PREs. The concentrations of both glucose (**Figure 3A**) and fructose (**Figure 3B**) were significantly reduced in PRE of potato plants 10 dpi with *M. persicae* at any level of inoculum (*P* < 0.05). An increasing number of aphids resulted in a significant dose-dependent reduction of glucose and fructose in the root exudates (Pearson’s correlation *r* = -0.772, *P* < 0.001 and *r* = -0.843, *P* < 0.001, glucose and fructose, respectively).

### Glucose and Fructose Induce Hatching of *G. pallida*

**FIGURE 4:**
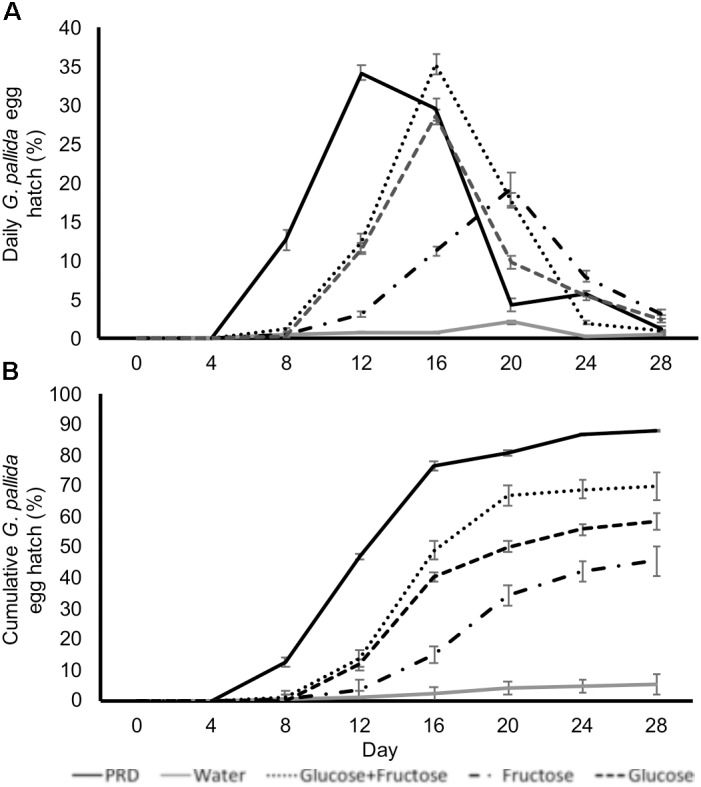
Daily **(A)** and cumulative **(B)** *Globodera pallida* percentage egg hatch from cysts treated with water, potato root exudate (PRE), 16.4 μg/ml glucose (Glu), and/or 35.0 μg/ml fructose (Fru). These concentrations reflect the concentrations detected in PRE. Values are means ± SEM from 10 replicates with five cysts per replicate.

In order to test if glucose and fructose directly stimulate hatching we incubated cysts in glucose and fructose solutions with concentrations equivalent to those detected in non-infested PRE. Treatment of *G. pallida* cysts with glucose and/or fructose induced egg hatch although peak hatching in sugar solutions occurred later than when cysts were treated with control PRE (**Figure 4A**). Total percentage egg hatch from cysts treated with sugars was greater than that from cysts treated with water but not as great as cysts treated with control PRE (**Figure 4B**; *P* < 0.01). Treatment with glucose and fructose combined resulted in significantly greater hatch than either single sugar but still significantly less than control PRE (**Figure 4**; *P* < 0.01).

### Supplementing Root Exudate From Aphid Infected Potato Plants With Glucose and Fructose Does Not Rescue *G. pallida* Hatch

**FIGURE 5:**
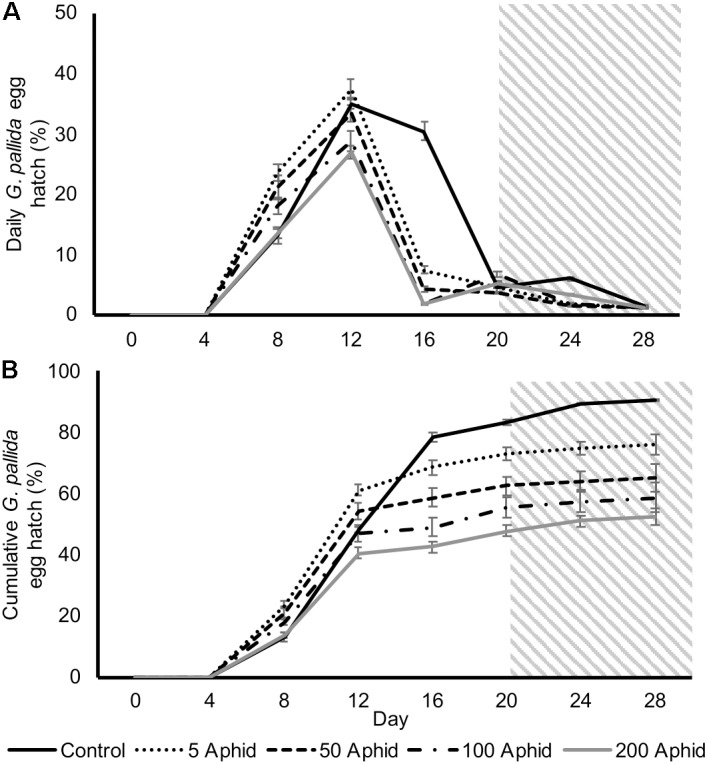
Daily **(A)** and cumulative **(B)** *Globodera pallida* percentage egg hatch from cysts treated with root exudate from control and *M. persicae* infested potato plants (days 0–20). Initial inoculums of 5, 50, 100, and 200 aphids were applied to the leaves of potato plants for 10 days before collection of exudate. Root exudate from infested plants was supplemented with glucose and fructose for treatments on days 20–28 (gray box) to equate to concentrations found in root exudate from non-infested potato plants (16.4 and 35.0 μg/ml, respectively). Values are means ± SEM from 10 replicates with five cysts per replicate.

In order to test whether the reduced hatching rate in aphid-infested PRE was due to a reduction in fructose and glucose, we supplemented those exudates with sufficient sugars to restore the concentrations found in non-infested PRE and used this as the replacement exudate at day 20. However, the reduced hatch rates were not rescued by supplementation of exudates with glucose and fructose, nor was total hatch significantly different (**Figures 5A,B**).

### Induced Expression of *Gpa-nep-1* Varies in Response to Hatching Stimulants

**FIGURE 6:**
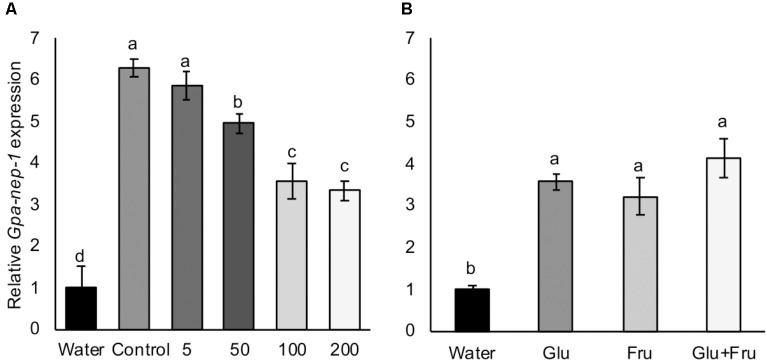
Expression of a neprilysin gene (*Gpa-nep-1*) by RT-qPCR in five *Globodera pallida* cysts treated or 8 days with water or **(A)** root exudate from potato plants inoculated with 0, 5, 50, 100, or 200 *Myzus persicae*. **(B)** 16.4 μg/ml glucose (Glu) and/or 35.0 μg/ml fructose (Fru). These concentrations reflect the concentrations detected in non-infested potato root exudate. Expression was normalized to *Elongation Factor 1*-α and presented relative to expression in cysts treated with water. Values are means ± SEM from four replicates with five cysts per replicate. Letters denote significant differences between treatments (*P* < 0.05).

A *Globodera* neprilysin gene has been detected as the first transcript to be upregulated in eggs treated with host root exudate (Duceppe et al., 2017), therefore we tested the expression of *Gpa-nep-1* in *G. pallida* eggs that had been incubated in non-infested control and aphid-infested PRE. There was a significant increase in the expression of *Gpa-nep-1* in unhatched *G. pallida* eggs 8 days post incubation in root exudates from non-infested control plants relative to eggs incubated in water (**Figure 6A**). Root exudates from aphid-infested potato plants significantly increased the expression of *Gpa-nep-1* in eggs but to a lower degree than non-infested control treatments (*P* < 0.05). There was also a significant increase in the expression of *Gpa-nep-1* in *G. pallida* eggs 8 days post incubation in glucose and/or fructose solutions relative to eggs in water (*P* < 0.01) (**Figure 6B**). Upregulation of *Gpa-nep-1* in response to the sugars was not as large as in eggs treated with PRE.

## Discussion

In this work, we demonstrate how the physiological response of the potato plant to attack by an above-ground herbivore, *Myzus persicae* can indirectly influence hatching of the soil-borne PPN, *Globodera pallida* through systemic changes in root exudates.

### Below-Ground Plant Responses to Aphid Infestation

The top-down effect of shoot herbivory on below-ground biomass is relatively undescribed compared to the more direct effects of root herbivores (Masters et al., 1993; Bardgett et al., 1998; Wu et al., 1999; Soler et al., 2005; Van Dam et al., 2005; Gratwick, 2012; McKenzie et al., 2016). We found that the root mass of potato plants was reduced in the presence of increasing inocula of *Myzus persicae* (**Figure 1**). Above-ground foliar herbivory may affect the roots, and therefore soil biotic communities by altering root carbon allocation and/or patterns of root exudation (Bardgett et al., 1998). Annuals, such as potato, do not store a high proportion of primary productivity in the root system and are therefore more likely to divert the products to the shoot to maintain foliar growth upon herbivory, thereby decreasing biomass of the root system (Mooney, 1972).

Aphids feed from plant phloem tissue via their stylets (Dixon and Kindlmann, 1998) by removing water, ions, sucrose, and free amino acids, which are major sources of carbon and nitrogen and vital for plant growth (Girousse et al., 2005). Aphids have been implicated in the translocation of sugars through their host plant (Hussain et al., 1974). Translocation of substances can occur from root to shoot and *vice versa*. A proteinaceous salivary sheath is released from the aphid stylet during feeding and can move long distances throughout the plant, causing deleterious effects (Madhusudhan and Miles, 1998; Miles, 1999; Burd, 2002). Pea aphid (*Acyrthosiphon pisum*) feeding on alfalfa stems strongly reduces carbon flux and initiates translocation of amino acids from roots, leaves, and sink tissues (Girousse et al., 2005). This translocation of assimilates from the roots has an effect of decreasing the root C:N ratio, thereby suggesting that plants allocate most productivity into regrowth of foliar tissues rather than root (Seastedt et al., 1988).

### Plant-Parasitic Nematode Responses to Root Exudation

The shift in root assimilates can modulate root exudation and can affect soil pathogens, such as rhizobacteria (Bardgett et al., 1998; Kim et al., 2016). Root exudates have traditionally been grouped into low- (amino acids, sugars, phenolics) and high (mucilage and proteins) molecular weight compounds. However, the complexity and chemical composition of root exudates from diverse plant species is unknown (Walker et al., 2003). Our results show that root exudates from aphid-colonized plants negatively affected nematode egg hatch, the initial stage of the life cycle, compared to exudates from non-infested control plants. Wounding of plants has been reported to elicit a defense response in roots (Savatin et al., 2014), however, all root exudates used in this study were prepared in the same way therefore the differences we observed between exudates reflect only the aphid infestations of the plants. Inhibition of hatch was positively correlated with size of aphid inoculum. This did not merely reflect the lower root mass of the aphid infested plants, which was taken into account during preparation of the exudate, suggesting that the composition of PRE may be indirectly changed as a result of the aphid feeding, in a dose-dependent manner. Aphid infestation has previously been reported to result in reduced infestation of *Arabidopsis* roots by pre-hatched J2s (Kutyniok and Müller, 2012). Compounds exuded by plant roots are known to stimulate the hatch of various cyst nematodes as well as affect stylet thrusting, attraction and transcription in other endoparasitic nematodes such as *Meloidogyne incognita* (Perry and Beane, 1989; Devine et al., 1996; Teillet et al., 2013).

### Effect of Aphid-Infestation on Potato Root Exudate Composition

Simple sugars are known to attract some nematode species and induce their stylet activity but this is not the case for *G. pallida*, possibly due to its selective host nature (Kamilova et al., 2006; Warnock et al., 2016). Root exudates from aphid-infested plants had a reduced concentration of glucose and fructose, but an active role of sugars in stimulating nematode hatch has not been previously described. Our study found that both glucose and fructose, at concentrations present in our PREs, were sufficient to induce hatching of *G. pallida*. The effect of sugars on hatching also correlated with an increase in *Gpa-nep-1* transcript within the eggs. In a previous study, a role in hatching has been proposed for this gene as it is the first *Globodera* transcript to be upregulated post-treatment with root exudates from host plants (Duceppe et al., 2017). This study reinforces that proposed link as it correlates the hatching ability of the exudate with expression levels of the gene.

The hatching stimulation of glucose and fructose and their effects on *Gpa-nep-1* expression infer hatching of *Globodera* in exudates from non-host plants, as previously observed for *G. ellingtonae* (Zasada et al., 2013). The variance of egg hatch between host root exudates suggests varying concentrations of hatching stimuli or hatching inhibitors. Confirming either of these factors could direct a new pathway for manipulation of exudates to protect plants from nematode attack, not only for *Globodera* spp., but also for other PPN.

### Effect of Aphid-Infestation on the Hatching of a Soil Borne Pathogen

Diminished hatching of *G. pallida* was partially recovered after treatment with root exudates from uninfested potato plants, indicating that the effect is reversible but cannot be fully recovered. The addition of sugars to exudates from aphid-infested plants did not increase their hatch stimulation. This suggests that as well as altering the sugar composition of exudant, aphid feeding may reduce the concentration of hatching stimuli and/or induce exudation of a factor/factors that can inhibit hatching. Exudates from control plants may reverse the effects of this compound, although not completely in some eggs, while sugars do not. Aphid feeding is known to induce systemic translocation and increased production of defense compounds, such as polyacetylenes (Wu et al., 1999), which can initiate defense pathways, such as the phytoalexin response (Flores et al., 1988) and play a role in resistance to nematodes (Veech, 1982). Additionally, genetic variation between individuals within a cyst could rationalize the portion of eggs that do not react to the hatching stimulant and are more susceptible to the inhibitory compound. Genetic variation is known to occur between individuals of *G. pallida* within a population (Eves-van den Akker et al., 2014) and could regulate the timeframe in which individual eggs hatch post-treatment with root exudate and in response to sugars. It would be of interest to determine variable loci, possibly *Gpa-nep-1*, in eggs with differential hatch under each treatment.

## Conclusion

Our data reveal the systemic effects of aphid colonization on potato plants and how the compositional shift of root exudate can negatively impact the hatch and gene transcription of the potato cyst nematode *G. pallida.* We have determined for the first time that the sugars fructose and glucose, present in root exudate, can induce hatching of a cyst nematode and we suggest the presence of an unidentified compound that may inhibit the hatching stimulus. This insight will assist efforts to establish what determines host status of a plant and underpin the production of plants that do not exude hatch-inducing compounds. Although *G. pallida* infects the host plant soon after roots emerge, while *M. persicae* colonize the plant once there is sufficient biomass above-ground (Emden, 1969), knowledge gained from the current study will be useful to inform management strategy for PPN, such as the beet and soybean cyst nematodes that can complete more than one generation in a cropping season (Alston and Schmitt, 1988).

## Author Contributions

GH, CB, CL, and PU designed the research. GH and CB performed the research. GH and CB analyzed the data. GH, CB, CL, and PU wrote the manuscript.

## Conflict of Interest Statement

The authors declare that the research was conducted in the absence of any commercial or financial relationships that could be construed as a potential conflict of interest.
